# Comparative Radiomics Analysis of Trabecular Bone Surrounding Dental Implants on Panoramic Radiographs in Smokers and Non-Smokers

**DOI:** 10.3390/diagnostics16213558

**Published:** 2026-10-29

**Authors:** Birkan Eyup Yilmaz, Busra Nur Gokkurt Yilmaz, Furkan Ozbey

**Affiliations:** 1 Department of Oral and Maxillofacial Surgery, Faculty of Dentistry, Giresun University, 28200 Giresun, Türkiye; 2 Department of Dentomaxillofacial Radiology, Faculty of Dentistry, Giresun University, 28200 Giresun, Türkiye; busranur581@hotmail.com; 3 Department of Dentomaxillofacial Radiology, Faculty of Dentistry, Afyonkarahisar Health Sciences University, 03030 Afyonkarahisar, Türkiye; furkanozbey3@gmail.com

**Keywords:** dental implants, smoking, radiomics, panoramic radiography, peri-implant bone, trabecular bone, texture analysis

## Abstract

**Background/Objectives**: Smoking is associated with adverse dental implant outcomes, but early smoking-related changes in peri-implant trabecular bone texture remain insufficiently characterized. This study evaluated longitudinal changes in panoramic radiomics features around dental implants in active smokers and non-smokers. **Methods**: This retrospective study included 47 patients and 110 implant sites, with 55 implant sites per group. Panoramic radiographs obtained at 0–7 days (T0), 4–6 weeks (T1), and 10–14 weeks (T2) after implant placement were analyzed. Twenty-eight first-order gray-level co-occurrence matrix (GLCM) and gray-level run length matrix (GLRLM) features were extracted; features with intraclass correlation coefficients ≥ 0.75 were included in mixed repeated-measures analyses. **Results**: Twenty-six features met the reliability threshold. Following BH-FDR correction across the 26 group-by-time interaction tests, significant interactions remained for GLCM Contrast (adjusted *p* = 0.010) and GLRLM Short Run Emphasis (SRE; adjusted *p* = 0.033). The Entropy interaction did not remain statistically significant after correction (adjusted *p* = 0.075). Contrast increased in smokers but remained relatively stable in non-smokers, whereas SRE decreased in smokers and increased in non-smokers. **Conclusions**: Smoking status was associated with divergent early peri-implant radiographic texture trajectories, particularly for Contrast and SRE. These findings indicate quantitative imaging differences rather than direct evidence of impaired osseointegration and warrant prospective validation.

## 1. Introduction

Smoking is an important modifiable risk factor associated with dental implant failure, reduced implant survival, and increased peri-implant marginal bone loss [1,2,3]. This association may be related not only to the presence of smoking but also to the level of exposure, with heavier smoking being linked to less favorable implant outcomes [4,5]. In addition, the intensity and duration of smoking exposure may influence implant-related outcomes, with higher smoking levels being associated with increased implant failure and peri-implant marginal bone loss [4,5]. Patient-related factors such as age and sex may also contribute to variability in bone response [6]. Accordingly, quantitative imaging assessment of early smoking-related changes in peri-implant bone may provide clinically relevant information.

The biological rationale for a potential effect of smoking on peri-implant bone healing is supported by its effects on systemic bone metabolism and local healing responses. Smoking has been associated with lower bone mineral density and greater bone loss, while preclinical studies of nicotine exposure have also reported adverse changes in osseointegration [6,7]. However, isolated nicotine exposure does not represent all constituents of tobacco smoke, and preclinical findings should not be directly equated with early peri-implant remodeling in humans [7].

Radiomics is an approach that converts intensity distributions and spatial texture patterns within medical images into quantitative features, thereby enabling image phenotyping beyond conventional visual assessment [8]. The Image Biomarker Standardisation Initiative (IBSI) has provided a common framework for standardizing radiomics feature definitions and calculation procedures to improve reproducibility across software platforms and analytical workflows [9]. PyRadiomics is an open-source platform that enables extraction of standardized feature families, including first-order, Gray-Level Co-occurrence Matrix (GLCM), and Gray-Level Run Length Matrix (GLRLM) features [10]. Studies evaluating radiomics in the jaw bones have demonstrated its applicability to dental and maxillofacial imaging while also highlighting methodological heterogeneity in image acquisition, segmentation, preprocessing, and feature extraction [11].

Fractal dimension and lacunarity analyses have long been used for quantitative assessment of trabecular bone on panoramic radiographs; however, differences in region-of-interest (ROI) selection and image-processing procedures contribute to heterogeneity across studies [12]. Panoramic radiography studies evaluating mandibular trabecular bone in smokers have reported lower fractal dimension or regional texture differences, although these findings have not been consistent across all measurement sites or radiographic parameters [13,14]. A recent study directly evaluating peri-implant regions also reported lower fractal dimension and higher lacunarity in smokers [15]. Nevertheless, the existing literature is predominantly based on cross-sectional designs and fractal/lacunarity-based measurements [13,14,15].

Evidence therefore remains limited regarding how peri-implant trabecular bone changes longitudinally during the early postoperative period according to smoking status when assessed at the same implant site using standardized first-order and matrix-based radiomics features [13,14,15]. The aim of this study was to compare changes in first-order, GLCM, and GLRLM radiomics features derived from panoramic radiographs of peri-implant trabecular bone from the immediate postoperative period to the third month between active smokers and individuals with no history of regular smoking.

## 2. Materials and Methods

### 2.1. Study Design and Ethics Approval

This retrospective, observational, comparative study was designed to longitudinally evaluate peri-implant trabecular bone in smokers and non-smokers using radiomics analysis of panoramic radiographs obtained during the postoperative period, at approximately 1 month and at approximately 3 months. The study protocol was approved by the Giresun University Medical Research Ethics Committee (Decision No. 11.06.2026/02), and the study was conducted in accordance with the principles of the Declaration of Helsinki.

### 2.2. Study Population and Sample Size

**Figure 1:**
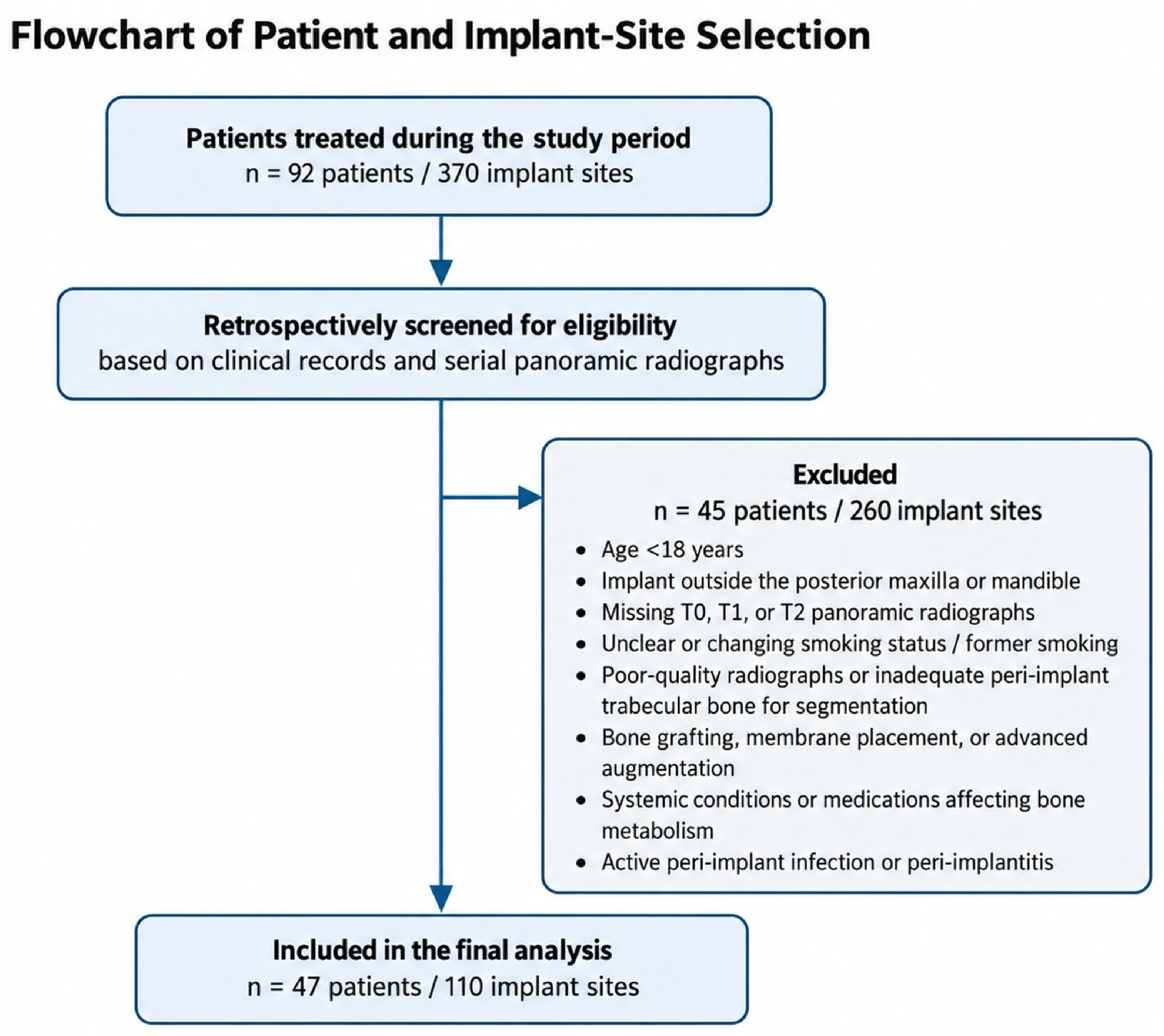
Flowchart of patient and implant-site selection for the final study cohort.

During the study period, 92 patients with 370 implant sites were retrospectively screened for eligibility. Of these, 47 patients with 110 implant sites fulfilled the predefined eligibility criteria and were included in the final analysis. The patient and implant-site selection process is summarized in Figure 1. A total of 55 implant sites were included in each smoking-status group. Each implant site was considered a separate anatomical unit of analysis because the peri-implant trabecular bone environment was site-specific, and each site was evaluated separately at T0, T1, and T2. Implants from different commercially available systems were included; however, all surgical procedures and postoperative follow-up were performed by the same surgeon using a standardized clinical protocol.

Sample size was calculated in G*Power version 3.1.9.4 using the “ANOVA: Repeated measures, within-between interaction” approach to evaluate the primary group × time interaction. Assuming a medium effect size (f = 0.25), α = 0.05, 95% statistical power, two groups, three repeated measurements, a correlation of 0.30 among repeated measures, and a nonsphericity correction of ε = 0.50, the minimum required total sample size was 100 implant sites. To account for potential data loss and exclusions, the target sample was increased by 10% to 110 implant sites.

### 2.3. Determination of Smoking Status

Smoking status was retrospectively obtained from patient files and medical history records. Individuals who smoked at least one cigarette per day during the evaluation period were classified as active smokers. The non-smoker group comprised individuals with no history of regular smoking.

### 2.4. Inclusion Criteria

The inclusion criteria were as follows: age ≥ 18 years; presence of a dental implant in the posterior maxilla or mandible; availability of postoperative, 4–6 weeks, and 10–14 weeks panoramic radiographs for the same implant site; clearly documented smoking status in the patient record; panoramic radiographs of diagnostic quality; and the presence of an evaluable peri-implant trabecular bone area suitable for radiomics segmentation.

### 2.5. Exclusion Criteria

Radiographs with marked motion blur, positioning errors, metal artifacts, or other image degradation were excluded. Patients were also excluded if bone grafting, membrane placement, or advanced augmentation procedures had been performed at implant placement; if they had osteoporosis, osteopenia, Paget disease, hyperparathyroidism, or another systemic condition affecting bone metabolism; or if they used bisphosphonates or other medications with a major effect on bone metabolism. Cases with active peri-implant infection or peri-implantitis were excluded, as were patients whose smoking status changed during follow-up, former smokers, and individuals whose smoking history could not be determined with sufficient accuracy.

### 2.6. Panoramic Radiograph Acquisition

Panoramic radiographs were acquired using a Hyperion X5 panoramic imaging system (MyRay, Imola, Bologna, Italy). Images were obtained using a tube voltage of 80 kVp and an exposure of 8 mA.

All images were acquired in DICOM format and anonymized before analysis. Three time points were evaluated for each implant site. T0 represented the postoperative image obtained within 0–7 days after implant placement; T1 represented the image obtained 4–6 weeks after implant placement; and T2 represented the image obtained 10–14 weeks after implant placement. Compressed files or images converted to other formats were excluded from analysis.

### 2.7. Region-of-Interest Selection and Segmentation

**Figure 2:**
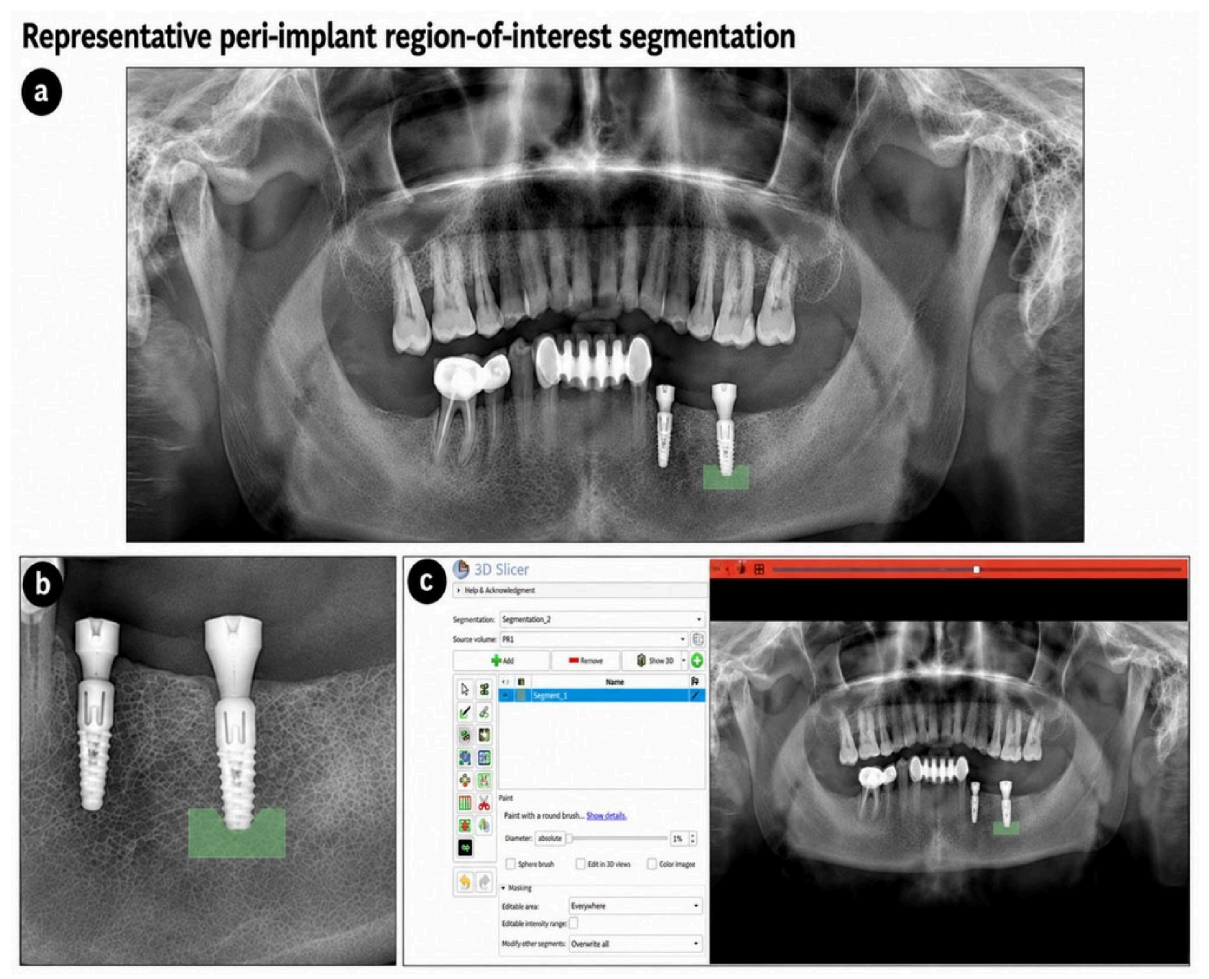
Representative segmentation of the peri-implant trabecular bone ROI positioned around the apical one-third of the implant. (**a**). Panoramic radiograph with the segmented peri-implant ROI. (**b**). Magnified ROI (**c**). ROI displayed in 3D Slicer.

At each implant site, a single continuous region of interest (ROI) representing peri-implant trabecular bone was manually segmented using 3D Slicer version 5.6.1. In all cases, the ROI was positioned around the apical one-third of the implant, beginning approximately 0.5 mm from the implant surface to minimize the influence of metal-related artifacts (Figure 2). The ROI was restricted to evaluable trabecular bone and excluded the implant body, cortical bone, the mandibular canal, the maxillary sinus floor, prominent anatomical superimpositions, and areas affected by metal artifacts. Thus, the apical peri-implant region was sampled consistently across all implant sites. All segmentations were performed by an experienced oral and maxillofacial radiologist.

The ROI size and shape were not predefined according to a fixed pixel dimension but were adapted to the available trabecular bone within the standardized apical peri-implant region. For longitudinal assessment, the implant position and surrounding anatomical landmarks were used as references to reproduce a comparable ROI location on the T0, T1, and T2 panoramic radiographs. Because the serial panoramic radiographs represented separate two-dimensional acquisitions and no image registration was performed, exact pixel-level correspondence of the same tissue across time points could not be guaranteed. Therefore, comparable rather than identical anatomical regions were evaluated longitudinally. The observer was blinded to patient smoking status during segmentation.

### 2.8. Radiomics Feature Extraction

Radiomics features were extracted using the SlicerRadiomics extension integrated into 3D Slicer and PyRadiomics version 3.1.0. No intensity normalization or resampling was applied to preserve the original gray-level distributions and spatial characteristics of the analyzed DICOM images. This approach was intended to avoid introducing additional interpolation-related changes into the extracted texture features. All panoramic radiographs at T0, T1, and T2 were acquired using the same imaging system (Hyperion X5, MyRay, Italy) and standardized exposure parameters (80 kVp and 8 mA) to minimize inter-examination variability. Nevertheless, the consistency of internal image-processing algorithms and pixel-intensity scaling across visits could not be independently verified. Therefore, residual variability related to image acquisition and processing cannot be excluded. A fixed-bin-width approach was used for gray-level discretization, with binWidth set to 25. Because panoramic radiographs are two-dimensional images, the force2D = True option was enabled.

GLCM features were calculated using a one-pixel neighborhood distance across all in-plane directions, and GLRLM features were likewise calculated across all in-plane directions. Only first-order Gray-Level Co-occurrence Matrix (GLCM) and Gray-Level Run Length Matrix (GLRLM) features derived from the original images were included in the analysis. Shape features were excluded because the segmentations were created to sample image texture from peri-implant trabecular bone rather than to delineate the true morphologic boundaries of an anatomic structure. Consequently, geometry-based shape metrics could have reflected the size and form of the investigator-defined segmentation rather than biological tissue morphology. Because the primary objective was to evaluate longitudinal changes in gray-level distribution and texture, the analysis was restricted to first-order, GLCM, and GLRLM features.

**Figure 3:**
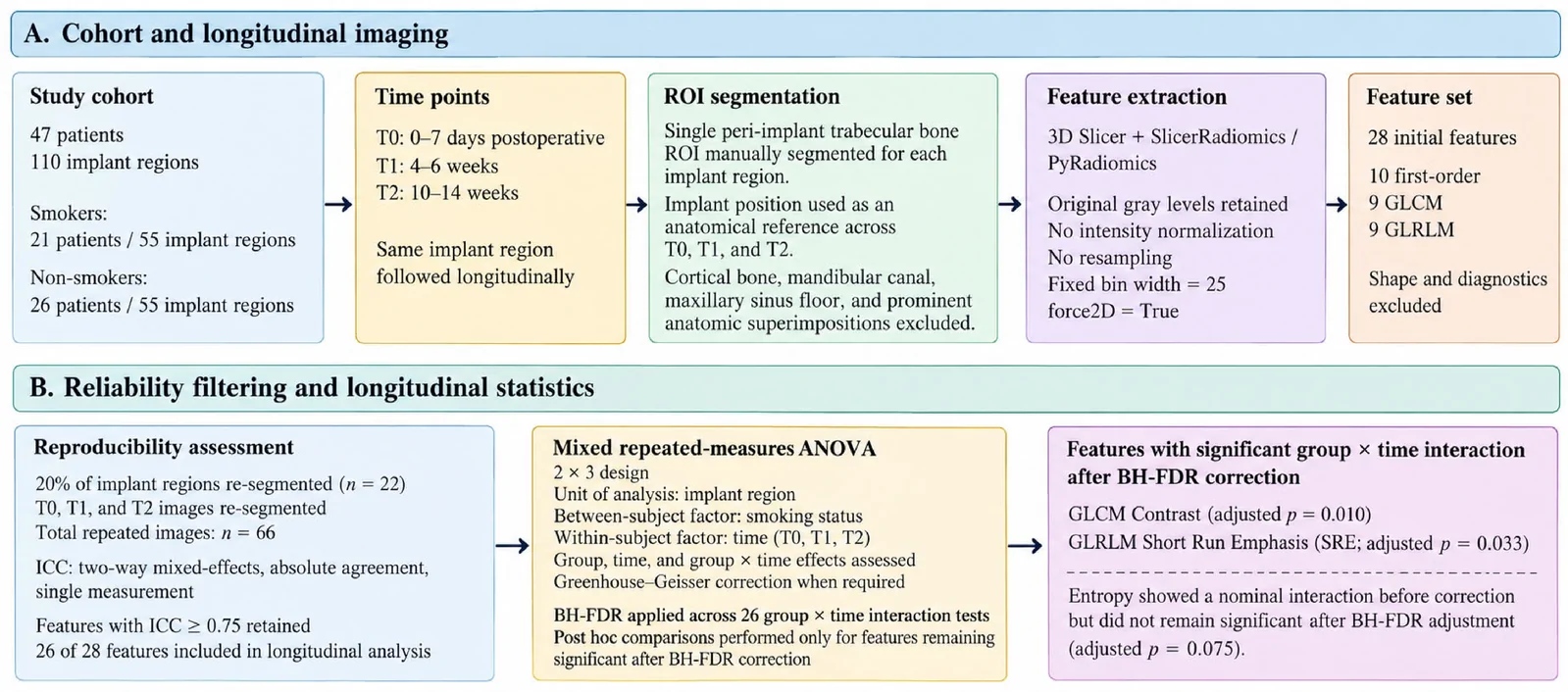
Summary flowchart of the analytical pipeline.

A total of 28 radiomics features were initially evaluated. First-order features included Energy, Entropy, Mean, Median, Variance, Skewness, Kurtosis, Mean Absolute Deviation, Interquartile Range, and Robust Mean Absolute Deviation. GLCM features included Contrast, Correlation, Cluster Shade, Cluster Prominence, Joint Energy, Joint Entropy, Inverse Difference Moment (IDM), Sum Average, and Sum Entropy. GLRLM features included Short Run Emphasis (SRE), Long Run Emphasis (LRE), Gray Level Non-Uniformity, Run Length Non-Uniformity, Gray Level Variance, Run Percentage, Run Variance, Low Gray Level Run Emphasis, and High Gray Level Run Emphasis (Figure 3).

### 2.9. Intraobserver Reliability

Twenty-two implant sites (20% of the total sample) were randomly selected for intraobserver reliability assessment. All 66 panoramic radiographs corresponding to these implant sites across the three time points (T0, T1, and T2) were re-segmented by the same observer after a two-week interval. For each radiomics feature, a single overall intraclass correlation coefficient (ICC) was calculated from the initial and repeated measurements of all 66 images, without separate analyses by time point or smoking status.

During the second evaluation, the observer was blinded to the initial segmentation and measurement results. The observer was also blinded to patient smoking status during segmentation.

Intraobserver reliability for each radiomics feature was assessed separately using the ICC based on a two-way mixed-effects model, absolute agreement, and single-measurement approach, with 95% confidence intervals (CIs). ICC values < 0.50 were interpreted as poor, 0.50–0.74 as moderate, 0.75–0.89 as good, and ≥0.90 as excellent reliability. Only radiomics features with ICC ≥ 0.75 were included in subsequent longitudinal analyses.

### 2.10. Statistical Analysis

Descriptive and statistical analyses were performed using IBM SPSS Statistics version 26.0 (IBM Corp., Armonk, NY, USA). Continuous variables were presented as mean ± standard deviation or median and interquartile range, according to their distribution; categorical variables were presented as number and percentage. Normality of continuous variables was assessed using the Shapiro–Wilk test.

Age and other continuous clinical variables were compared between groups using the independent-samples *t*-test when the normality assumption was met and the Mann–Whitney U test otherwise. Categorical variables, including sex and implant location, were evaluated using the chi-square test; Fisher’s exact test was used when expected cell frequencies were insufficient.

All radiomics features meeting the ICC ≥0.75 threshold in the intraobserver reliability analysis were included in the longitudinal analysis. For each radiomics feature, a 2 × 3 mixed-design repeated-measures analysis of variance was performed, with smoking status as the between-subject factor and time (T0, T1, and T2) as the within-subject factor. The main effects of group and time and the group × time interaction were evaluated separately in each model.

The sphericity assumption was examined using Mauchly’s test. When sphericity was violated, degrees of freedom were corrected using the Greenhouse-Geisser method. Effect sizes for main effects and interactions were expressed as partial eta squared (ηp^2^); values of approximately 0.01, 0.06, and 0.14 were interpreted as small, medium, and large effects, respectively.

To account for multiple testing across the 26 radiomics features, the Benjamini–Hochberg false discovery rate (BH-FDR) correction was applied to the 26 group × time interaction *p*-values obtained from the mixed-design repeated-measures ANOVA. Both unadjusted and BH-FDR-adjusted *p*-values were reported, and an adjusted *p*-value < 0.05 was considered statistically significant for the primary interaction analyses.

For radiomics features retaining a statistically significant group × time interaction after BH-FDR correction, post hoc pairwise comparisons were performed to identify the source of the interaction. Within each smoking-status group, T0–T1, T0–T2, and T1–T2 were compared; between-group differences were also evaluated separately at T0, T1, and T2. The nine post hoc comparisons for each selected feature were treated as a separate family, and BH-FDR correction was applied within each family. Both raw and adjusted *p*-values were reported.

## 3. Results

### 3.1. Demographic and Clinical Characteristics

A total of 47 patients (21 smokers and 26 non-smokers) and 110 dental implants (55 implant sites in each group) were included (Table 1). The number of implants contributed per patient ranged from 1 to 6 in smokers and from 1 to 4 in non-smokers. In the smoker group, 4 patients contributed one implant, 10 contributed two implants, 1 contributed three implants, 4 contributed four implants, and 2 contributed six implants. In the non-smoker group, 11 patients contributed one implant, 6 contributed two implants, 4 contributed three implants, and 5 contributed four implants. No statistically significant between-group differences were observed in age, sex distribution, or implant location (*p* > 0.05).

### 3.2. Intraobserver Reliability

The intraobserver ICC values calculated for the 22 re-segmented implant sites are presented in Table 2. Of the 28 radiomics features, 26 had ICC values ≥ 0.75, whereas 2 had ICC values < 0.75. Skewness (ICC = 0.74) and Kurtosis (ICC = 0.73) were below the predefined reliability threshold and were therefore excluded from subsequent analyses.

### 3.3. Longitudinal Changes in Radiomics Features

All 26 radiomics features meeting the ICC ≥ 0.75 threshold were analyzed using mixed repeated-measures ANOVA. Table 3 presents the *p*-values for the group effect, time effect, and group × time interaction, together with BH-FDR-adjusted *p*-values for the 26 interaction tests. Before correction, significant group × time interactions were observed for Entropy (*p* = 0.009), GLCM Contrast (*p* < 0.001), and GLRLM Short Run Emphasis (SRE; *p* = 0.002). Following BH-FDR correction, only Contrast (adjusted *p* = 0.010) and SRE (adjusted *p* = 0.033) retained statistical significance, whereas Entropy was no longer statistically significant (adjusted *p* = 0.075).

Descriptive statistics, F statistics, and effect sizes for the three features showing nominally significant group × time interactions (unadjusted *p* < 0.05) are presented in Table 4. However, only Contrast and SRE remained statistically significant after BH-FDR correction.

Although Entropy showed a numerically greater decrease in non-smokers than in smokers from T0 to T2 (Δ = 0.33 vs. 0.14), its group × time interaction did not remain statistically significant after BH-FDR correction (adjusted *p* = 0.075). Contrast progressively increased in smokers (T0: 28.4; T2: 34.8), whereas no statistically significant longitudinal change was observed in non-smokers. SRE showed a slight increase in non-smokers but decreased in smokers.

Mauchly’s test indicated a violation of the sphericity assumption for Contrast (χ^2^(2) = 7.84, *p* = 0.020); therefore, Greenhouse-Geisser-corrected results were reported (ε = 0.88).

### 3.4. Post Hoc Comparisons

Post hoc comparisons were performed for Contrast and SRE, the two radiomics features that retained statistically significant group × time interactions after BH-FDR correction across the 26 primary tests. Nine pairwise comparisons were evaluated for each feature, with BH-FDR correction applied separately within each family of comparisons (Table 5).

For Contrast, smokers showed a continuous and significant increase from T0 onward (T0–T2: pFDR = 0.0045), whereas no statistically significant change was observed between any pair of time points in non-smokers. The between-group difference was significant at T1 (pFDR = 0.0120) and T2 (pFDR = 0.0045), but not at T0 (pFDR = 0.0570).

SRE changed over time in both groups, but in opposite directions. SRE decreased in smokers (T0–T2: pFDR = 0.0045) and increased in non-smokers (T0–T2: pFDR = 0.0180). The between-group difference was significant at T1 (pFDR = 0.0150) and T2 (pFDR = 0.0045), but not at T0 (pFDR = 0.0579).

## 4. Discussion

The principal finding of this study was that GLCM Contrast (adjusted *p* = 0.010) and GLRLM Short Run Emphasis (SRE; adjusted *p* = 0.033) exhibited significantly different early peri-implant radiographic texture trajectories between smokers and non-smokers after BH-FDR correction across 26 interaction tests. Although Entropy showed a nominally significant group × time interaction, this finding did not remain statistically significant after correction (adjusted *p* = 0.075). These findings suggest that smoking may be associated with a distinct quantitative imaging phenotype during early peri-implant trabecular bone remodeling. However, because these features do not directly measure histologic osseointegration or bone loss, they should primarily be interpreted as radiographic texture changes.

The study most directly comparable with our findings is the peri-implant fractal analysis reported by Sevimay et al. [15]. The authors evaluated 108 men with posterior mandibular implants, including 56 smokers and 52 non-smokers, and reported lower fractal dimension and higher lacunarity in smokers across all mesial, distal, and apical peri-implant regions [15]. In the present study, between-group divergence also became more pronounced over follow-up: GLCM Contrast increased from 28.4 ± 8.2 to 34.8 ± 9.7 in smokers but decreased from 24.1 ± 7.5 to 22.9 ± 6.8 in non-smokers, whereas GLRLM SRE decreased from 0.812 ± 0.045 to 0.781 ± 0.051 in smokers and increased from 0.835 ± 0.039 to 0.852 ± 0.035 in non-smokers. Although the measured parameters are mathematically distinct, both studies indicate that peri-implant trabecular image patterns differ according to smoking status. Extending the cross-sectional findings of Sevimay et al., our longitudinal data suggest that this difference may not represent only a static group characteristic but may also involve divergent texture trajectories during early healing.

Findings from panoramic radiography studies of mandibular trabecular bone in smokers have not been entirely homogeneous. Basavarajappa et al. evaluated 225 individuals and found a lower fractal dimension in smokers than in controls (1.4781 ± 0.0505 vs. 1.5043 ± 0.0597; *p* = 0.02), whereas lacunarity did not differ significantly between groups [13]. In the study by Çelik and Arslan, which included 56 individuals and evaluated eight mandibular ROIs, differences in fractal dimension were observed in only two ROIs, while radiomorphometric indices did not differ between groups [14]. Similarly, Azman and Taşsöker found no significant fractal-dimension difference in two mandibular ROIs among 55 smokers and 55 non-smokers, although fractal dimension tended to decrease with increasing smoking exposure [16]. In our study, only Contrast and SRE showed statistically significant group × time interactions after BH-FDR correction across the 26 radiomics features, whereas the nominal interaction observed for Entropy did not remain significant. In particular, the increase in Contrast among smokers together with its decrease in non-smokers, and the opposite directional changes in SRE, suggest that smoking-related image phenotypes may be selective rather than uniformly expressed across all texture measures. This pattern is conceptually consistent with previous fractal studies in which differences emerged only in specific ROIs or metrics, and it also accords with the dental fractal-analysis literature emphasizing the influence of ROI selection and image-processing procedures [12].

The potential clinical relevance of this early texture divergence should be interpreted in the context of the long-term effects of smoking on implant outcomes. Wach et al. followed 2196 peri-implant radiographic samples from 768 patients for 5 years and reported different bone-texture characteristics together with less favorable clinical outcomes in smokers; by year 5, implant loss occurred in 6.74% of smokers versus 2.87% of non-smokers, and marginal bone loss was also greater in the smoking group [17]. Similarly, Mustapha et al. associated smoking with an approximately 2.4-fold higher risk of implant failure and an additional 0.58 mm of marginal bone loss [1], while the meta-analysis by Fan et al., comprising 59,246 implants, reported an OR of 2.59 for early implant failure [2]. Calciolari et al. reported an average of 0.64 mm greater crestal bone loss in smokers [3]. In the present study, the between-group difference in GLCM Contrast reached 11.9 by the third month, and SRE also showed clear separation between groups; however, implant loss, peri-implantitis, and long-term marginal bone loss were not evaluated. The present radiomics findings can therefore be considered within the broader context of a smoking-associated effect reported in long-term clinical studies, but they should not be interpreted as evidence that early changes in Contrast or SRE are precursors of subsequent implant failure. Establishing such a relationship would require prospective correlation of these same radiomics features with long-term clinical outcomes.

The dose of smoking exposure may represent another factor contributing to differences across studies. In the meta-analysis by Naseri et al., smoking more than 20 cigarettes per day was associated with an implant-failure risk of RR = 2.45 at the implant level and RR = 4.00 at the patient level [4]. Afshari et al. likewise showed that greater daily cigarette consumption may be associated with greater peri-implant marginal bone loss, with a pooled difference of approximately 0.58 mm between individuals smoking more than 10 cigarettes per day and non-smokers [5]. In the present study, active smoking was defined as at least one cigarette per day, and smokers were not stratified according to exposure dose. Nevertheless, the clearly divergent longitudinal trajectories of Contrast and SRE suggest that smoking status may generate a measurable signal in early peri-implant texture. Grouping light and heavy smokers together may, however, have attenuated a true dose–response relationship. Future studies directly examining associations between pack-years or daily cigarette consumption and the magnitude of radiomics feature change may therefore provide additional insight.

The opposite directional changes observed in Contrast and SRE are biologically noteworthy. The association of smoking with adverse systemic bone effects, together with evidence that nicotine exposure can negatively affect osseointegration in preclinical models, provides a plausible biological context for different early remodeling responses between groups [6,7]. From a radiomics perspective, GLCM Contrast reflects local gray-level differences between neighboring pixels, whereas SRE reflects the relative weighting of short, homogeneous gray-level runs [9,10]. In the present study, Contrast increased by approximately 23% in smokers while decreasing by approximately 5% in non-smokers; conversely, SRE decreased in smokers and increased in non-smokers, indicating opposite changes in peri-implant texture organization during early follow-up. This divergent trajectory suggests that smoking may be related not only to the baseline imaging phenotype but also to the manner in which texture reorganizes during healing. Nevertheless, because Contrast and SRE do not directly measure trabecular thickness, bone mineral density, or bone-implant contact, labeling these changes as “bone resorption” or “impaired osseointegration” would exceed the evidence provided by the present data.

The use of quantitative texture features derived from panoramic radiographs for dental bone assessment is expanding. A systematic review of jaw-bone radiomics identified GLCM, first-order, and GLRLM among the most frequently used feature families [11]. In the peri-implant setting, Efeoglu et al. evaluated 439 peri-implant regions from 144 patients and reported an AUC of 0.86 for their best-performing machine-learning model [18]. Konishi et al. achieved an AUC of 0.903 in the test set using panoramic-radiograph features from 32 patients with medication-related osteonecrosis of the jaw and 57 controls [19], while Anandan et al. demonstrated multiple trabecular texture differences using GLCM, fractal, and strut-based measures in 272 panoramic radiographs [20]. Uzun et al. compared mandibular cortical index groups using radiomics in 159 panoramic radiographs and reported between-group differences in several features, including Entropy (*p* = 0.001), GLCM Correlation (*p* < 0.001), and GLRLM Gray Level Variance (*p* = 0.001); however, the C2-C3 distinction did not remain significant after correction for multiple comparisons [21]. In our study, only two of the 26 radiomics features, Contrast and SRE, demonstrated significant group × time interactions after BH-FDR correction, with both features showing opposite longitudinal trajectories between smokers and non-smokers. This parallel supports the potential of panoramic radiomics to quantify differences in bone imaging phenotypes while also indicating that not all radiomics features provide equivalent biological or clinical information. Accordingly, the present findings extend previous cross-sectional panoramic radiomics studies by demonstrating that radiomics may also be used to track temporal changes within the same anatomic region [22].

The principal contribution of this study is the evaluation of early temporal changes at the same implant sites rather than classification of smoking-related peri-implant bone differences at a single time point. Most previous smoking studies have provided cross-sectional comparisons based on fractal dimension or lacunarity [13,14,15,16], whereas the present study tracked first-order, GLCM, and GLRLM features across three consecutive time points. This approach allowed baseline group differences to be distinguished from divergence developing during follow-up. Although Entropy exhibited a greater descriptive decrease in non-smokers than in smokers, its group × time interaction did not survive correction for multiple testing (adjusted *p* = 0.075). Therefore, this observation should be considered exploratory. In contrast, GLCM Contrast (adjusted *p* = 0.010) and GLRLM SRE (adjusted *p* = 0.033) retained significant group × time interactions after BH-FDR correction, providing stronger statistical evidence of divergent early peri-implant radiographic texture trajectories between smokers and non-smokers.

Several limitations should be considered. This was a retrospective single-center study, and detailed smoking-exposure data, including daily cigarette consumption, smoking duration, and pack-years, were unavailable. Although all procedures and follow-up assessments were performed by the same surgeon using a standardized protocol, different implant systems were included, and implant dimensions and surface characteristics were not analyzed. Another limitation is that some patients contributed multiple implants to the analysis. In addition, panoramic radiography is two-dimensional and sensitive to projection geometry, and the 3-month follow-up did not allow assessment of long-term clinical outcomes. Future multicenter studies with larger cohorts, detailed smoking-exposure data, and long-term clinical validation are warranted.

## 5. Conclusions

In conclusion, active smoking was associated with a different temporal trajectory of radiomics texture features derived from panoramic radiographs during the early peri-implant period. This divergence was most evident for GLCM Contrast (adjusted *p* = 0.010) and GLRLM SRE (adjusted *p* = 0.033), both of which remained statistically significant after BH-FDR correction, indicating different early peri-implant radiographic texture trajectories between smokers and non-smokers. These findings extend the selective radiographic differences reported in previous studies of smoking and peri-implant bone to a longitudinal radiomics framework. Nevertheless, these features should be interpreted as quantitative radiographic markers of early remodeling differences rather than direct measures of biological damage. Prospective studies linking these features with long-term clinical outcomes are needed to determine whether they have prognostic value.

## Figures and Tables

**Table 1:** Demographic and clinical characteristics of patients.

Variable	Smokers	Non-Smokers	*p*-Value
**Patient level**	(*n* = 21)	(*n* = 26)	
**Age (years), mean ± SD**	42.3 ± 8.7	39.8 ± 9.2	0.341 ^a^
**Sex (Female/Male)**	10/11	12/14	0.923 ^b^
**Implant level**	(*n* = 55)	(*n* = 55)	
**Location (Maxilla/Mandible)**	28/27	31/24	0.574 ^b^

^a^ Independent-samples *t*-test; ^b^ chi-square test.

**Table 2:** Intraobserver reliability results for radiomics features.

Feature	ICC	95% CI
**First-order features**		
**Energy**	0.94	0.90–0.97
**Entropy**	0.93	0.89–0.96
**Mean**	0.96	0.93–0.98
**Median**	0.95	0.92–0.97
**Variance**	0.88	0.81–0.93
**Skewness †**	0.74	0.60–0.85
**Kurtosis †**	0.73	0.63–0.86
**Mean Absolute Deviation**	0.91	0.85–0.95
**Interquartile Range**	0.90	0.84–0.94
**Robust Mean Abs. Deviation**	0.89	0.82–0.94
**GLCM features**		
**Contrast**	0.92	0.87–0.95
**Correlation**	0.91	0.86–0.95
**Cluster Shade**	0.85	0.76–0.91
**Cluster Prominence**	0.83	0.73–0.90
**Joint Energy**	0.93	0.89–0.96
**Joint Entropy**	0.94	0.90–0.97
**IDM**	0.90	0.84–0.94
**Sum Average**	0.95	0.92–0.97
**Sum Entropy**	0.92	0.87–0.95
**GLRLM features**		
**Short Run Emphasis (SRE)**	0.93	0.89–0.96
**Long Run Emphasis (LRE)**	0.91	0.85–0.95
**Gray Level Non-Uniformity**	0.88	0.80–0.93
**Run Length Non-Uniformity**	0.94	0.90–0.97
**Gray Level Variance**	0.87	0.79–0.92
**Run Percentage**	0.92	0.87–0.96
**Run Variance**	0.86	0.78–0.92
**Low Gray Level Run Emphasis**	0.84	0.74–0.91
**High Gray Level Run Emphasis**	0.90	0.84–0.94

ICC, intraclass correlation coefficient; CI, confidence interval. Two-way mixed-effects model, absolute agreement, single measurement. † ICC < 0.75; these features were excluded from subsequent analyses.

**Table 3:** Mixed-ANOVA results for the 26 radiomics features meeting the ICC threshold, including BH-FDR-adjusted group × time interaction *p*-values.

Feature	Group *p*	Time *p*	Group × Time *p*	BH-FDR-Adjusted Group × Time *p*
**First-order**				
**Energy**	0.124	0.038	0.287	0.579
**Entropy**	<0.001	<0.001	0.009	0.075
**Mean**	0.412	0.076	0.541	0.586
**Median**	0.387	0.082	0.496	0.586
**Variance**	0.067	0.045	0.312	0.579
**Mean Absolute Deviation**	0.089	0.051	0.264	0.579
**Interquartile Range**	0.156	0.063	0.378	0.586
**Robust Mean Abs. Dev.**	0.134	0.058	0.341	0.586
**GLCM**				
Contrast *	0.001	0.007	<0.001	0.010
**Correlation**	0.045	0.112	0.187	0.579
**Cluster Shade**	0.523	0.345	0.612	0.612
**Cluster Prominence**	0.478	0.289	0.567	0.590
**Joint Energy**	0.098	0.034	0.215	0.579
**Joint Entropy**	0.112	0.041	0.198	0.579
**IDM**	0.067	0.156	0.423	0.586
**Sum Average**	0.345	0.089	0.456	0.586
**Sum Entropy**	0.087	0.042	0.178	0.579
**GLRLM**				
Short Run Emphasis (SRE) *	0.002	0.042	0.002	0.033
**Long Run Emphasis (LRE)**	0.034	0.067	0.156	0.579
**Gray Level Non-Uniformity**	0.256	0.134	0.478	0.586
**Run Length Non-Uniformity**	0.078	0.045	0.312	0.579
**Gray Level Variance**	0.189	0.098	0.389	0.586
**Run Percentage**	0.045	0.056	0.134	0.579
**Run Variance**	0.167	0.123	0.456	0.586
**Low Gray Level Run Emph.**	0.312	0.234	0.534	0.586
**High Gray Level Run Emph.**	0.078	0.067	0.245	0.579

* Statistically significant group × time interaction after BH-FDR correction (adjusted *p* < 0.05). BH-FDR correction was applied across the 26 group × time interaction tests. Greenhouse–Geisser-corrected values are reported for Contrast.

**Table 4:** Detailed results for radiomics features showing nominally significant group × time interactions before multiple-testing correction.

	Group	T0 (Mean ± SD)	T1 (Mean ± SD)	T2 (Mean ± SD)	Effect	F	*p*	η_p_^2^
**Entropy**	Smoker	5.82 ± 0.41	5.75 ± 0.38	5.68 ± 0.35	Group	F(1,108) = 14.2	<0.001	0.116
	Non-smoker	5.54 ± 0.37	5.38 ± 0.34	5.21 ± 0.31	Time	F(2,216) = 18.7	<0.001	0.148
					G × T	F(2,216) = 4.85	0.0087	0.043
**Contrast**	Smoker	28.4 ± 8.2	31.5 ± 9.1	34.8 ± 9.7	Group	F(1,108) = 11.8	<0.001	0.098
	Non-smoker	24.1 ± 7.5	23.8 ± 7.2	22.9 ± 6.8	Time	F(1.76,190) = 5.42 †	0.0072	0.048
					G × T	F(1.76,190) = 8.93 †	<0.001	0.076
**SRE**	Smoker	0.812 ± 0.045	0.798 ± 0.048	0.781 ± 0.051	Group	F(1,108) = 9.67	0.0024	0.082
	Non-smoker	0.835 ± 0.039	0.841 ± 0.037	0.852 ± 0.035	Time	F(2,216) = 3.21	0.0423	0.029
					G × T	F(2,216) = 6.14	0.0025	0.054

† Greenhouse-Geisser corrected (Mauchly’s test of sphericity: χ^2^(2) = 7.84, *p* = 0.020, ε = 0.88). After BH-FDR correction across the 26 interaction tests, only Contrast (adjusted *p* = 0.010) and SRE (adjusted *p* = 0.033) remained significant, whereas Entropy did not (adjusted *p* = 0.075). The *p*-values presented in this table are unadjusted.

**Table 5:** (A). Post hoc comparisons for GLCM Contrast. (B). Post hoc comparisons for GLRLM Short Run Emphasis (SRE).

Comparison	Mean Difference	Raw *p*	BH-FDR *p*
(A)			
**Smoker: T0–T1**	−3.1	0.015	0.0338 *
**Smoker: T0–T2**	−6.4	<0.001	0.0045 *
**Smoker: T1–T2**	−3.3	0.021	0.0378 *
**Non-smoker: T0–T1**	0.3	0.721	0.7210
**Non-smoker: T0–T2**	1.2	0.352	0.4526
**Non-smoker: T1–T2**	0.9	0.413	0.4646
**T0: Smoker vs. Non-smoker**	4.3	0.038	0.0570
**T1: Smoker vs. Non-smoker**	7.7	0.004	0.0120 *
**T2: Smoker vs. Non-smoker**	11.9	<0.001	0.0045 *
(B)			
**Smoker: T0–T1**	0.014	0.045	0.0579
**Smoker: T0–T2**	0.031	0.001	0.0045 *
**Smoker: T1–T2**	0.017	0.028	0.0504
**Non-smoker: T0–T1**	−0.006	0.312	0.3120
**Non-smoker: T0–T2**	−0.017	0.008	0.0180 *
**Non-smoker: T1–T2**	−0.011	0.052	0.0585
**T0: Smoker vs. Non-smoker**	−0.023	0.042	0.0579
**T1: Smoker vs. Non-smoker**	−0.043	0.005	0.0150 *
**T2: Smoker vs. Non-smoker**	−0.071	<0.001	0.0045 *

FDR, Benjamini–Hochberg false discovery rate correction; applied within the family of nine comparisons for each feature. * *p* < 0.05.

## Data Availability

The datasets used and/or analyzed during the current study are available from the corresponding author on reasonable request.
